# High Vancomycin MIC and Complicated Methicillin-Susceptible *Staphylococcus aureus* Bacteremia

**DOI:** 10.3201/eid1706.101037

**Published:** 2011-06

**Authors:** Jose Maria Aguado, Rafael San-Juan, Antonio Lalueza, Francisca Sanz, Joaquin Rodríguez-Otero, Carmen Gómez-Gonzalez, Fernando Chaves

**Affiliations:** Author affiliations: University Hospital 12 de Octubre, Madrid, Spain

**Keywords:** methicillin-susceptible Staphylococcus aureus, bacteria, MSSA, bacteremia, vancomycin, Spain, dispatch

## Abstract

We conducted a retrospective study of 99 patients with methicillin-suseptible *Staphylococcus aureus* catheter-related bacteremia in which vancomycin MIC was determined by Etest. High vancomycin MIC (>1.5 μg/mL) was the only independent risk factor for development of complicated bacteremia caused by methicillin-susceptible *S. aureus* (odds ratio 22.9, 95% confidence interval 6.7–78.1).

Several studies have established a relationship between high vancomycin MIC and a worse prognosis for patients with bacteremia caused by methicillin-resistant *Staphylococcus aureus* (MRSA) (*1**–**5*). However, to our knowledge, the role that a high vancomycin MIC could play in the clinical course of a patient with methicillin-susceptible *S. aureus* (MSSA) bacteremia, has not been investigated, although a high vancomycin MIC has been also reported for strains of MSSA (*6*).

## The Study

We retrospectively determined the MIC of vancomycin for the first MSSA blood culture isolate from a cohort of 99 adult patients with catheter-related bacteremia. These patients were consecutively evaluated from January 2002 through December 2004 (mean follow-up 3 years) in University Hospital 12 de Octubre in Madrid, Spain, a 1,000-bed university medical center.

We determined methicillin and vancomycin susceptibility by using broth microdilution according to Clinical Laboratory Standards Institute methods. Vancomycin MIC of was determined under blinded conditions by Etest in the first isolate by using a 0.5 McFarland inoculum streaked evenly with a swab onto Mueller-Hinton agar plates (*7*).

Complicated bacteremia was defined by one of the following events occurring after the first episode of bacteremia: 1) development of endocarditis, septic thrombophlebitis (defined by persistent MSSA bacteremia at least 72 hours after initiation of active antimicrobial drugs + documented thrombi), arthritis, spondylitis, as well as end-organ hematogenous spread of infection to other locations; or 2) infection involving vascular or osteoarticular prostheses (excluding intravascular catheter) not removed within 4 days. We also calculated the crude death rate in the first 30 days after the first positive blood culture (30-day mortality) and mortality rate attributable to *S. aureus* bacteremia (attributable death rate).

The Student unpaired *t* test was used to compare continuous variables, the Mann-Whitney U test to compare continuous variables with a nonnormal distribution, and the Fisher exact test to compare proportions. All statistical tests were 2-tailed and the threshold of statistical significance was p<0.05. To analyze the risk factors for development of complicated bacteremia, we performed a multivariate forward stepwise logistic regression model including all the clinically relevant variables with a p value of <0.05 and possible confounding factors with a p value of <0.1 detected in the univariate analysis (SPSS software version 15.0, SPSS, Chicago, IL, USA).

All 99 MSSA strains were susceptible to vancomycin (MIC <2 µg/mL) by the broth microdilution method. Our data showed that, in 23/99 (23.2%) strains, MICs of vancomycin were >1.5 µg/mL by Etest (range 1.5–1.7 µg/mL).

Comparative data of patients with or without a high vancomycin MIC MSSA strain are shown in Table 1. The incidence of severe sepsis/septic shock was similar in both groups (21.7% vs. 14.5%; p = 0.69), but patients with high vancomycin MIC strains had complicated bacteremia more frequently (78.3% vs. 13.2%; p<0.0001). Attributable death rate was higher in patients with high vancomycin MIC strains with a difference that nearly achieves statistical significance (17.4% vs. 3.9%; p = 0.08).

**Table 1:** Differential characteristics of patients with bacteremia caused by MSSA strains with a MIC to vancomycin >1.5 µg/mL compared with <1.5 µg/mL by Etest, Hospital 12 de Octubre, Madrid, Spain, January 2002–December 2004*

Characteristic	MIC <1.5, n = 76	MIC >1.5, n = 23	p value
Mean age, y (SD)	63.55 (16.7)	62.9 (18.8)	0.87
M/F, %	69.7/30.3	56.5/43.5	0.36
Co-morbidity Charlson Index, mean (SD)	2.76 (2.7)	3.4 (3.7)	0.4
Previous valvular prosthesis	1 (1.3)	2 (8.7)	0.23
Other previous endovascular prosthesis	4 (5.3)	1 (4.3)	0.7
Previous osteoarticular prosthesis	3 (3.9)	0	0.79
Previous renal failure requiring hemodialysis	7 (9.2)	4 (17.4)	0.47
Type of IV catheter as the source of bacteremia			
Peripheral line	34 (44.7)	9 (39.1)	0.71
Transitory central catheter	34 (44.7)	10 (43.5)	0.82
Permanent central catheter	8 (10.6)	4 (17.4)	0.76
Vancomycin MIC of the first MSSA isolate, median (range)	1.2 (0.5–1.4)	1.5 (1.5–1.7)	<0.0001
Initial treatment with glycopeptides	46 (60.5)	18 (78.3)	0.19
Initial treatment with antistaphylococcal β-lactams†	20 (26.3)	5 (21.7)	0.87
Initial treatment with non–β-lactam anti-staphylococcal agents‡	7 (9.2)	0	0.29
Delay in initiation of active antibiotic treatment, d,§ mean (SD)	0.85 (1.06)	1.3 (1.6)	0.14
Duration of antibiotic treatment, d, mean (SD)	13.4 (8.24)	18.6 (12)	0.07
Prompt IV catheter removal¶	62 (81.6)	17 (73.9)	0.45
Conservative IV catheter management#	4 (5.3)	2 (8.7)	0.32
Development of severe sepsis/septic shock	11 (14.5)	5 (21.7)	0.69
Complicated bacteremia	10 (13.2)	18 (78.3)	<0.0001
Septic thrombophlebitis	5 (6.6)	8 (34.9)	0.002
Endocarditis	3 (3.9)	4 (17.3)	0.08
Osteoarticular	2 (2.6)	2 (8.7)	0.48
Pulmonary emboli	0	2 (8.7)	0.08
Other	0	2 (8.7)	0.08
Crude 30-day death rate	8 (10.5)	6 (26.1)	0.13
Attributable death rate	3 (3.9)	4 (17.4)	0.083

*Values are no. (%) except as indicated. MSSA, methicillin-susceptible *Staphylococcus aureus*; IV, intravenous. †Antistaphylococcal β-lactams refers to parenteral cloxacillin, cefazolin, amoxicillin-clavulanate, piperacillin-tazobactam, or imipenem/meropenem. ‡Including non–β-lactam antibiotics with in vitro activity against MSSA (mostly levofloxacin, moxifloxacin or, clindamycin). §Delay since isolation of MSSA in blood cultures. ¶Removal of catheter in the first 48 hours since isolation of MSSA in blood cultures. #Catheter kept at least 7 days since isolation of MSSA in blood cultures.

Comparative data between the 28 patients in whom complicated bacteremia developed and the remaining cohort are shown in Table 2. The percentage of isolates with vancomycin MIC >1.5 µg/mL was significantly higher in patients with complicated bacteremia (18/28 [64.3%] vs. 5/71 [7%]; p<0.0001). Initial treatment with glycopeptides was more frequent in patients in whom complicated bacteremia developed (82.1% vs 57.7%; p = 0.042). Among the 64 patients treated initially with glycopeptides, the rate of complicated bacteremia was significantly higher in patients with high vancomycin MIC isolates (15/18 [83.3%] vs. 8/46 [17.4%]; p<0.0001), as occurred in the 25 patients treated initially with β-lactams, (3/5 [60%] vs. 2/20 [10%]; p = 0.064). Vancomycin MIC >1.5 µg/mL was the only variable independently related to the risk for complicated bacteremia (OR 22.9, 95% confidence interval 6.7–78.1) in the multivariate analysis.

**Table 2:** Comparative analysis of 99 patients with complicated vs. noncomplicated MSSA bacteremia, Hospital 12 de Octubre, Madrid, Spain, January 2002–December 2004*

Variable	Noncomplicated MSSA, n = 71	Complicated MSSA, n = 28	p value
Mean age, y (SD)	63.9 (17.4)	62 (16)	0.6
M/F, %	69/31	60.7/39.3	0.8
Co-morbidity Charlson Index, mean (SD)	2.92 (2.4)	2.93 (3.5)	0.9
Previous valvular prosthesis	1 (1.4)	2 (7.1)	0.39
Other previous endovascular prosthesis	4 (5.6)	1 (3.6)	0.9
Previous ostheoarticular prosthesis	2 (2.8)	1 (3.6)	0.8
Previous renal failure requiring hemodyalisis	8 (11.3)	3 (10.7)	0.8
Type of IV catheter as the source of bacteremia			
Peripheral line	32 (45.1)	11 (39.3)	0.7
Transitory central catheter	30 (42.2)	14 (50)	0.8
Permanent central catheter	9 (12.7)	3 (10.7)	0.9
Vancomycin MIC for the first MSSA isolate, median (range)	1.2 (0.5–1.7)	1.5 (1.0–1.7)	<0.0001
Vancomycin MIC >1.5 µg/mL for the first MSSA isolate	5 (7)	18 (64.3)	<0.0001
Initial treatment with glycopeptides	41 (57.7)	23 (82.1)	0.042
Initial treatment with antistaphylococcal β-lactams†	20 (28.2)	5 (17.9)	0.42
Initial treatment with non–β-lactam antistaphylococcal agents‡	7 (9.9)	0	0.19
Delay in initiation of active antibiotic treatment, d,§ mean (SD)	0.92 (1.3)	1.07 (1)	0.8
Delay >24 h at the start of effective antibiotics§	28 (39.4)	14 (53.6)	0.2
Duration of antibiotic treatment, d, mean (SD)	12.77 (8)	19.39 (11.4)	0.002
Prompt IV catheter removal¶	58 (81.7)	21 (75)	0.65
Conservative IV catheter management#	5 (7)	1 (3.6)	0.8
Development of severe sepsis/septic shock	9 (12.7)	7 (25)	0.23
Days of follow-up, mean (SD)	502 (441)	462 (463)	0.77
Crude 30-day death rate	9 (12.7)	5 (17.9)	0.9
Attributable death rate	3 (4.2)	4 (14.3)	0.18

*Values are no. (%) except as indicated. MSSA, methicillin-susceptible *Staphylococcus aureus*; IV, intravenous. †Antistaphylococcal β-lactams refer to parenteral cloxacillin, cefazolin, amoxicillin-clavulanate, piperacillin-tazobactam, or imipenem/meropenem. ‡Including non–β-lactam antibiotics with in vitro activity against MSSA (mostly levofloxacin, moxifloxacin, or clindamycin). §Delay since isolation of MSSA in blood cultures. ¶Removal of catheter in the first 48 h since isolation of MSSA in blood cultures. #Catheter kept at least 7 days since isolation of MSSA in blood cultures.

## Conclusions

The aim of our study was to evaluate whether vancomycin MIC has any influence on the death rates and outcomes of patients with catheter-related MSSA bacteremia. We chose a MIC >1.5 µg/mL as interpretive criteria for diminished susceptibility on the basis of the reported treatment failure for infections caused by organisms who have exhibited this level of vancomycin MIC (*5**,**8*). A first relevant finding of our study was the relatively high incidence of high vancomycin MIC among MSSA strains producing bacteremia (23.2%), a result similar to the percentage found for MRSA strains in our hospital (*9**,**10*).

Although a previous study found that vancomycin MICs for MSSA strains recovered from hemodialysis-dependent patients with bacteremia who had been treated with vancomycin did not seem to be related to their clinical outcomes (*11*), recently published in vitro data suggest that isolates of *S. aureus* with high vancomycin MICs could be less susceptible to cloxacillin or daptomycin (*6*). Our data showed that patients with MSSA bacteremia caused by strains with high vancomycin MIC were not related to a higher rate of severe sepsis/septic shock development but were associated with a higher rate of complicated bacteremia. In fact, complicated bacteremia was related to a vancomycin MIC >1.5 µg/mL but not with other factors such as age, acquisition of infection, severity of underlying disease, or catheter management, which was confirmed in the multivariate analysis.

The initial treatment most frequently associated with complicated bacteremia, in patients with and without high vancomycin MIC, was the use of glycopeptides alone or followed by antistaphylococcal β-lactams. A possible explanation for this finding is that the first hours of antibiotic treatment are crucial to avoid complications. Nevertheless, in our opinion the greatest risk for complicated bacteremia related to strains of *S. aureus* with high vancomycin MIC should not only be attributed to the fact that these strains are more resistant to vancomycin because patients infected with strains with high vancomycin MIC that were initially treated with β-lactams also had a clear tendency to develop more complicated bacteremia. Nevertheless, the scarce number of patients who were treated initially with β-lactams limit our results. We hypothesize that certain structural modifications might also occur in the cell wall of strains with high vancomycin MIC, including a thicker cell wall as it has been described in MRSA (*12*). Thickness of the cell wall should not only hinder the action of vancomycin, but also the arrival to the target (penicillin binding proteins of β-lactams). If this hypothesis is correct, a vancomycin MIC of 1.5–2 µg/mL in MSSA could be not only a marker of poor response to vancomycin but also a subrogate marker of suboptimal response to β-lactams and even pathogenicity, as has been recently suggested in MRSA isolates (*13*).

Some limitations of this study deserve specific consideration. For most patients a treatment schedule including glycopeptides and β-lactams was used, so it is difficult to analyze the role played by each antimicrobial drug. All our strains had a vancomycin MIC <2 µg/mL, so we do not know which would be the outcome of MSSA bacteremia caused by more resistant strains. We did not specifically test clonality of strains with high vancomycin MIC because we had previously demonstrated that MSSA strains isolated from patients with bacteremia at our institution (which coincided with most of the strains included in the present study) were polyclonal (*14*). Finally, some important variables such as the previous use of vancomycin or vancomycin serum levels were not included in the analysis.

A high level of resistance to vancomycin is related with the development of complicated complicated bacteremia caused by MSSA, independent of the type of initial antibiotic treatment. Failure of glycopeptides does not appear to be the unique factor for the development of complicated bacteremia in patients with high vancomycin MIC isolates, and therefore intrinsic characteristics of these strains could also explain MSSA’s pathogenic role in the development of complicated bacteremia.

## References

[1] Lodise TP, Graves J, Evans A, Graffunder E, Helmecke M, Lomaestro BM, Relationship between vancomycin MIC and failure among patients with methicillin-resistant *Staphylococcus aureus* bacteremia treated with vancomycin. Antimicrob Agents Chemother. 2008;52:3315–20. 10.1128/AAC.00113-08 18591266 PMC2533486

[2] Moise PA, Sakoulas G, Forrest A, Schentag JJ. Vancomycin in vitro bactericidal activity and its relationship to efficacy in clearance of methicillin-resistant *Staphylococcus aureus* bacteremia. Antimicrob Agents Chemother. 2007;51:2582–6. 10.1128/AAC.00939-06 17452488 PMC1913284

[3] Sakoulas G, Moise-Broder PA, Schentag J, Forrest A, Moellering RC Jr, Eliopoulos GM. Relationship of MIC and bactericidal activity to efficacy of vancomycin for treatment of methicillin-resistant *Staphylococcus aureus* bacteremia. J Clin Microbiol. 2004;42:2398–402. 10.1128/JCM.42.6.2398-2402.2004 15184410 PMC427878

[4] Schwaber MJ, Wright SB, Carmeli Y, Venkataraman L, DeGirolami PC, Gramatikova A, Clinical implications of varying degrees of vancomycin susceptibility in methicillin-resistant *Staphylococcus aureus* bacteremia. Emerg Infect Dis. 2003;9:657–64. 12781004 10.3201/eid0906.030001 PMC3000153

[5] Soriano A, Marco F, Martinez JA, Pisos E, Almela M, Dimova VP, Influence of vancomycin minimum inhibitory concentration on the treatment of methicillin-resistant *Staphylococcus aureus* bacteremia. Clin Infect Dis. 2008;46:193–200. 10.1086/524667 18171250

[6] Pillai SK, Wennersten C, Venkataraman L, Eliopoulos G, Moellering R, Karchmer A. Development of reduced vancomycin susceptibility in methicillin-susceptible *Staphylococcus aureus.* Clin Infect Dis. 2009;49:1169–74. 10.1086/605636 19769538

[7] Walsh TR, Bolmstrom A, Qwarnstrom A, Ho P, Wootton M, Howe RA, Evaluation of current methods for detection of staphylococci with reduced susceptibility to glycopeptides. J Clin Microbiol. 2001;39:2439–44. 10.1128/JCM.39.7.2439-2444.2001 11427551 PMC88167

[8] Tenover FC, Moellering RC Jr. The rationale for revising the Clinical and Laboratory Standards Institute vancomycin minimal inhibitory concentration interpretive criteria for *Staphylococcus aureus.* Clin Infect Dis. 2007;44:1208–15. 10.1086/513203 17407040

[9] Lalueza A, Chaves F, San Juan R, Daskalaki M, Otero JR, Aguado JM. Is high vancomycin minimum inhibitory concentration a good marker to predict the outcome of methicillin-resistant *Staphylococcus aureus* bacteremia? J Infect Dis. 2010;201:311–2. 10.1086/649572 20034343

[10] Lalueza A, Chaves F, San Juan R, Daskalaki M, López-Medrano M, Lizasoain M, Less severity but higher risk of late complications in methicillin-resistant *Staphylococcus aureus* bacteremia with a vancomycin MIC ≥1.5 µg/mL. In: Microbiology ASF, editor. 49th Interscience Conference on Antimicrobials Agents and Chemotherapy. San Francisco: American Society for Microbiology; 2009.

[11] Stryjewski ME, Szczech LA, Benjamin DK Jr, Inrig JK, Kanafani ZA, Engemann JJ, Use of vancomycin or first-generation cephalosporins for the treatment of hemodialysis-dependent patients with methicillin-susceptible *Staphylococcus aureus* bacteremia. Clin Infect Dis. 2007;44:190–6. 10.1086/510386 17173215

[12] Cui L, Ma X, Sato K, Okuma K, Tenover FC, Mamizuka EM, Cell wall thickening is a common feature of vancomycin resistance in *Staphylococcus aureus.* J Clin Microbiol. 2003;41:5–14. 10.1128/JCM.41.1.5-14.2003 12517819 PMC149586

[13] Peleg AY, Monga D, Pillai S, Mylonakis E, Moellering RC Jr, Eliopoulos GM. Reduced susceptibility to vancomycin influences pathogenicity in *Staphylococcus aureus* infection. J Infect Dis. 2009;199:532–6. 10.1086/596511 19125671 PMC3750955

[14] Chaves F, Garcia-Martinez J, de Miguel S, Sanz F, Otero JR. Epidemiology and clonality of methicillin-resistant and methicillin-susceptible *Staphylococcus aureus* causing bacteremia in a tertiary-care hospital in Spain. Infect Control Hosp Epidemiol. 2005;26:150–6. 10.1086/502519 15756885

